# Isisekelo Sempilo study protocol for the effectiveness of HIV prevention embedded in sexual health with or without peer navigator support (Thetha Nami) to reduce prevalence of transmissible HIV amongst adolescents and young adults in rural KwaZulu-Natal: a 2 × 2 factorial randomised controlled trial

**DOI:** 10.1186/s12889-022-12796-8

**Published:** 2022-03-07

**Authors:** Glory Chidumwa, Natsayi Chimbindi, Carina Herbst, Nonhlanhla Okeselo, Jaco Dreyer, Thembelihle Zuma, Theresa Smith, Jean-Michel Molina, Thandeka Khoza, Nuala McGrath, Janet Seeley, Deenan Pillay, Frank Tanser, Guy Harling, Lorraine Sherr, Andrew Copas, Kathy Baisley, Maryam Shahmanesh

**Affiliations:** 1 Africa Health Research Institute, Mtubatuba, KwaZulu-Natal South Africa; 2 UCL Institute for Global Health, 3rd Floor Mortimer Market Centre, Capper Street, London, WC1E 6JP UK; 3 University of KwaZulu-Natal, Durban, South Africa; 4 Department of Infectious Diseases, Hospitals Saint-Louis and Lariboisière, Paris, France; 5 University of Southampton, Southampton, UK; 6 London School of Hygiene & Tropical Medicine, London, UK; 7 Lincoln University, London,, UK; 8 University of the Witwatersrand, Johannesburg, South Africa; 9 Harvard T.H. Chan School of Public Health, Boston, USA

**Keywords:** Peer navigator, HIV prevention, Community-based care, Contraception, Pre-Exposure prophylaxis

## Abstract

**Background:**

Antiretroviral therapy (ART) through universal test and treat (UTT) and HIV pre-exposure prophylaxis (PrEP) substantially reduces HIV-related mortality, morbidity and incidence. Effective individual-level prevention modalities have not translated into population-level impact in southern Africa due to sub-optimal coverage among adolescents and youth who are hard to engage. We aim to investigate the feasibility, acceptability, and preliminary population level effectiveness of HIV prevention services with or without peer support to reduce prevalence of transmissible HIV amongst adolescents and young adults in KwaZulu-Natal.

**Methods:**

We are conducting a 2 × 2 factorial trial among young men and women aged 16–29 years, randomly selected from the Africa Health Research Institute demographic surveillance area. Participants are randomly allocated to one of four intervention combinations: 1) Standard of Care (SOC): nurse-led services for HIV testing plus ART if positive or PrEP for those eligible and negative; 2) Sexual and Reproductive Health (SRH): Baseline self-collected vaginal and urine samples with study-organized clinic appointments for results, treatment and delivery of HIV testing, ART and PrEP integrated with SRH services; 3) Peer-support: Study referral of participants to a peer navigator to assess their health, social and educational needs and provide risk-informed HIV prevention, including facilitating clinic attendance; or 4) SRH + peer-support.

The primary outcomes for effectiveness are: (1) the proportion of individuals with infectious HIV at 12 months and (2) uptake of risk-informed comprehensive HIV prevention services within 60 days of enrolment. At 12 months, all participants will be contacted at home and the study team will collect a dried blood spot for HIV ELISA and HIV viral load testing.

**Discussion:**

This trial will enable us to understand the relative importance of SRH and peer support in creating demand for effective and risk informed biomedical HIV prevention and preliminary data on their effectiveness on reducing the prevalence of transmissible HIV amongst all adolescents and youth.

**Trial registration:**

Trial Registry: clincialtrials.gov. ClinicalTrials.gov Identifier NCT04532307. Registered: March 2020.

## Background

Despite advances in HIV treatment and talk of “end of AIDS”, HIV-related morbidity and mortality remains the largest health problem in South Africa: 7.7 million people in the country are living with HIV and there are 200,000 new infections per year, the highest numbers in the world [1]. Young people bear the brunt of new HIV infections and the socioeconomic fallout of the HIV pandemic [2, 3]. The demographic shift and doubling in number of young people over the next twenty years underscores the urgency of developing scalable models of delivering HIV prevention alongside treatment [4].

HIV incidence among women aged 20–24 in KwaZulu-Natal, while decreasing, remains unacceptably high at 5.8/100 person-years (PY) [5]. The ambitious scale-up of combination behavioural and structural interventions to reduce HIV in adolescent girls and young women (AGYW) (DREAMS) did not accelerate declines in HIV incidence [6], with recent reductions in incidence rather explained by male partners’ access to HIV prevention and treatment [7]. South Africa has been at the forefront of developing and implementing substantial advances in biomedical HIV prevention tools [8]. These include: HIV point of care tests (POCT) and self-tests [9, 10]; the use of daily oral tenofovir/emtricitabine and long acting cabotegravir (an injectable drug) for pre-exposure prophylaxis (PrEP) which reduce HIV acquisition by up to 90%, far exceeding that obtained by condoms in real-world settings [11–13]; and HIV treatment with ART that eliminates onward transmission of HIV [14].

Unfortunately, these highly effective biomedical preventions are not consistently reaching the most vulnerable: young people aged 16–30 years [15, 16]. In HIV test and treat trials fewer than one third of this age-group who were diagnosed during the trial went on to access HIV care [17, 18]. They thus failed to receive the health benefits of ART and remained able to pass on the virus. In parallel, primary health care systems, particularly in rural settings, are failing to reach young people with contraception, resulting in high levels of teenage pregnancy [19].

HIV programmes have not successfully tackled psychosocial needs of youth: in the Africa Health Research Institute’s (AHRI) demographic surveillance area in rural KwaZulu-Natal, over 85% of school-leavers are unemployed [20] and there are high levels of common mental disorders (CMD) which increase with age (rising to 32% of those aged 20–22) [21]. Efforts to reduce structural vulnerabilities through multi-level interventions (social asset building and parenting interventions) was less successful in reaching older adolescents, those out of school, and those with CMD were less likely to access structural interventions [6].

A 2016 population-based study of 15–24-year-olds in rural KwaZulu-Natal found a very high burden of sexually transmitted infections (STI) (20% of women and 10% of men had a curable infections) [22] and an extremely high incidence of teenage pregnancy 6.4/100PY. The same study also found that home-based self-sampling and treatment for STIs was acceptable and desirable for young people [23], and sometimes forms the basis for creating demand for care and prevention of HIV within community-based sexual and reproductive health (SRH) services.

There is growing evidence on the effectiveness of community-based HIV care. A global meta-analysis found that community health worker HIV care delivery significantly improved HIV viral suppression, which also reduces sexual transmission [24]. The DOART trial in KZN showed that community-based care (in which people are tested for HIV in the community and started on ART without visiting a clinic) was superior to facility-based HIV treatment (in which people had to attend a clinic for treatment) in suppressing HIV viral load [25]. Community-based approaches, when integrated with wider psychosocial care, foster social networks and norms that endorse HIV care and improve sustainable development goals [3, 26–29]. Integrated, community-based approaches are particularly important for adolescents [2], e.g., a peer-led intervention integrated with psychosocial support for adolescents living with HIV in Zimbabwe was the first to show improvements in adolescent HIV viral suppression [27, 30, 31].

Evidence for peer-led interventions to support HIV prevention is emerging [2, 28, 32]. A systematic review of peer-based interventions with young people found improvements in knowledge, sexual behaviour, and condom use across 12 studies [33]. The Empower trial combined participatory learning approaches to reduce gender-based violence with PrEP support in SA. It was valued by young women but had limited impact on PrEP retention [32]. Building on this evidence we used community-based participatory research to develop the *Thetha Nami* (Talk to Me) intervention*.* Men and women aged 18–30 years were selected by community leaders as potential peer-navigators and took part in participatory intervention development workshops (2016–2018). The co-created *Thetha Nami* included area-based peer-navigators providing safe spaces and community advocacy, using a structured assessment tool to tailor peer mentorship, and referral to health and social services. We found that this community-based delivery of HIV care and prevention with peer support was acceptable and feasible [34].

We hypothesise that integrating tailored HIV prevention and care (including universal test and treat (UTT) and PrEP) with youth-led services to improve adolescents and young adults’ sexual and reproductive health will improve uptake of HIV prevention and contraception, and therefore reduce HIV incidence and improve SRH outcomes.

### Objectives

The overarching goal of our research programme is to arrest the HIV epidemic and reduce its negative impact on young people in South Africa. We expect to achieve this by rapidly developing and testing the efficacy and efficiency of risk-informed, tailored HIV care and prevention interventions (including PrEP and UTT) that address demand, improve access, and support adherence in adolescents and young people. Our aim in this 2 × 2 factorial randomised controlled trial (RCT) is to integrate advances in participatory intervention development, process evaluation, and multi-arms within a common platform trial to evaluate the hypothesis that innovative and tailored HIV prevention interventions developed with and for young people will optimise models to deliver HIV prevention and care. The trial’s primary objectives are to measure the effectiveness of these interventions that have been developed with young people to reduce sexually transmissible HIV, and to increase uptake of risk-informed HIV prevention in young people in rural South Africa.

### Methods

#### Trial design

**Fig. 1:**
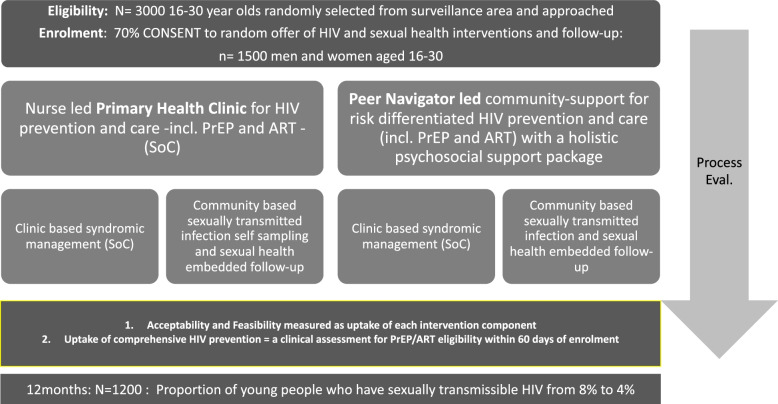
Isisekelo Sempilo 2x2 factorial RCT Diagram

The effectiveness of the interventions on sexually transmissible HIV viral load and the uptake of risk-informed PrEP/ART-based HIV prevention will be tested with a 2 × 2 randomised factorial trial among young people aged 16–29 years. Consenting individuals are randomised to one of 4 arms, to receive one of two delivery models (clinic referral only (SOC) or peer navigator support), with or without a comprehensive SRH package (Fig. 1). This design will allow us to efficiently measure the effect of different delivery models, and of offering a comprehensive SRH package, on a number of HIV care and prevention outcomes.

### Study setting and participants

This trial is embedded in AHRI’s HIV prevention programme based in the uMkhanyakude district in rural KwaZulu-Natal, South Africa [35]. The AHRI demographic surveillance area has a population of around 140,000, including > 20,000 16–29-year-olds. The study area is mostly rural, and poor compared with other parts of South Africa, with high levels of unemployment (over 85% of young adults aged 20–24 are unemployed), and a high prevalence of HIV. In 2017, AHRI implemented the ClinicLink system, where data collection clerks capture electronically the date and reason for attendance for all consenting individuals attending any of the 11 clinics in the surveillance area; residents are linked to their surveillance identification number at the time of the clinic visit. ClinicLink will allow us to measure linkage of individuals to HIV care and use of contraceptive services.

We use the AHRI demographic surveillance as a sampling frame to select a random sample of 3000 men and women aged 16–29 years old, stratified by sex, and invite them to participate in the study. Individuals are eligible to enrol in the study if they are between 16–29 years old, resident in the surveillance area, willing and able to provide informed consent, willing to be followed up at 12 months, and willing to provide a dried blood spot (DBS) for anonymous HIV testing and HIV viral load measurement at 12 months. Based on previous studies in this setting, we expect that 2000 will be contactable and eligible, and 1500 (75%) will enrol.

### Interventions

During the formative phase of this trial, we worked closely with a team of 57 peer navigators (who have undergone training on confidentiality, research ethics, and HIV prevention PrEP/ART/SRH clinical topics), social scientist facilitators and in liaison with the Department of Health to identify the intervention components to test in this trial [36]:

#### Enhanced Standard of Care (SOC) – clinic-based standard HIV prevention and treatment package

**Fig. 2:**
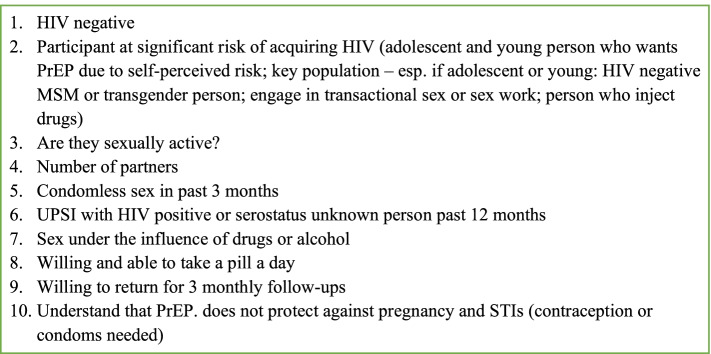
South Africa National Guidelines PrEP screening tool

All enrolled participants are provided with a barcoded referral slip and an appointment time to attend a clinic of their choice. Clinical services are provided by study nurses in two primary health clinics (PHC) situated in a busy commercial area adjacent to the AHRI surveillance area with adolescent and youth friendly services, and two mobile clinics that visit fixed sites across the more remote areas of the surveillance area once every 2 weeks. All clinic attendees (irrespective of trial arm) are offered HIV counselling and POCT, and immediate initiation of ART if positive or PrEP if negative and eligible according to South African National PrEP guidelines (Fig. 2). If the participant agrees to PrEP/ART initiation, the nurse issues them with a month’s supply of generic tenofovir disoproxil fumarate and emtricitabine (TDF/FTC) or ART, on the same day. This is followed by a telephone follow-up 7 days after initiating PrEP/ART to complete a standard symptom screen for adverse effects; participants are asked to attend the clinic if indicated. Participants are asked to attend the clinic at months 1, 2, 6, 9 and 12, as per national guidelines, for repeat HIV testing (if on PrEP), laboratory HIV viral load or ELISA confirmation if needed, safety bloods, clinic-based counselling and adherence support and PrEP/ART refills. All clinic attendees are also offered family planning support and syndromic management for STIs, partner notification documentation and, if male and HIV-negative, referral to voluntary male medical circumcision (VMMC), as per South African National Department of Health Guidelines.

#### Intervention 1 – *Thetha Nami* peer navigator support

participants who are randomised to this intervention will be offered the support of a named pair of *Thetha Nami* peer-navigators who work in their area. *Thetha Nami* are 54 area-based men and women aged 18–30 years, post matriculation, who are employed to provide a package of health and social support to young people aged 16–29 years living in their areas. Participants will be offered the peer navigators’ contact details and told that, unless they object, their contact details will be passed onto the peer navigators who will attempt to contact them within 7 days. The peer navigators will use a brief questionnaire to identify the participant’s needs and will provide the participant with any support that is required, including support in accessing the clinical service to which they have chosen to be referred, and, for those who start PrEP/ART, support as part of their individualised adherence plan, and for refills, appointment scheduling and reminders.

#### Intervention 2 – *Isisekelo Sempilo* with SRH

Participants who are randomised to this intervention will provide samples for sexually transmitted infection (STI) testing to the research assistants at enrolment (3–4 self-sampled vaginal swabs or first-catch urine for women; first-catch urine for men). These samples are sent to AHRI laboratories to be processed for gonorrhoea, chlamydia and trichomonas. Participants will be provided with a clinic appointment at a study clinic of their choosing (see enhanced SOC above) in 7 days to receive the results of their STI tests. They will be informed that if they default the appointment and any of the results are positive a nurse or research assistant will attempt to contact them by phone or in person to ensure that they and their partners receive the appropriate therapy to treat the infection. During the clinic appointment they will receive tailored sexual health counselling with an emphasis on tackling the multiple health-related behaviours that will affect fertility and sexual pleasure (STIs, mental health, alcohol, diet and exercise); assessment of fertility desire and as appropriate preconception or contraception counselling; a choice of contraception and condoms. HIV POCT will be offered as part of sexual health, counselling with PrEP to stay negative and ART in the context of staying well and Undetectable = Uninfectious (U = U). In addition to the SOC procedures, adherence support in this arm will include HIV viral load result-informed additional adherence and U = U counselling before PrEP/ART refills.

### Outcomes

#### Primary outcomes

There are three co-primary outcomes: (1) effectiveness of the intervention in reducing the prevalence of sexually transmissible HIV; (2) effectiveness of different components of the intervention to improve demand for universal risk-informed HIV prevention and treatment; and (3) acceptability and feasibility of recruiting and following up adolescent and youth participants in an HIV prevention trial platform.

We will measure sexually transmissible HIV as the proportion of participants who are HIV positive and have a detectable HIV viral load at 12 months after enrolment, defined as having an HIV viral load of ≥ 400 copies/mL. This outcome captures the effect of the intervention on both incident HIV and untreated HIV. We argue that if our intervention is successful there will be fewer cases of young people who acquire HIV, and if they have or acquire HIV, they will be identified and started on treatment. In both situations, the number of individuals with unsuppressed (transmissible) HIV virus will be reduced. We will measure demand for risk-informed HIV prevention and treatment as the proportion of participants who link to clinical services for HIV testing and PrEP/ART counselling within 60 days of enrolment. We will define acceptability of the trial as > 75% consent to participate in the trial, and feasibility as obtaining a HIV ELISA and viral load result in > 75% of participants 12 months after enrolment.

#### Secondary outcomes

Secondary outcomes of the trial include effectiveness of the intervention in improving the following: (1) treatment outcomes in participants living with HIV; (2) provision of risk informed HIV prevention, including PrEP; (3) sexual and reproductive health in all participants; and (4) mental health in all participants. These outcomes will be measured by: (1) the proportion of participants living with HIV who start treatment, the time from enrolment to HIV testing, and the time from testing positive to starting treatment; (2) the proportion of HIV-negative participants who are eligible and start PrEP, and the proportion of initially HIV-negative participants with a new HIV diagnosis at 12 months; (3) the proportion of all participants who are using contraception at 12 months, the proportion with an STI diagnosed at 12 months, and the proportion of women who become pregnant by 12 months; (4) the proportion of participants screening positive for a depression on the PHQ-9 questionnaire at 12 months.

### Sample size

With 2000 eligible and assuming that 75% consent to being randomised, we can estimate acceptability of randomisation with a precision of ± 1.9%. With 1500 enrolled, assuming that 80% of those enrolled are followed up at 12 months, we can estimate this proportion (i.e. feasibility of collecting outcome data in enrolled participants) with a precision of ± 2.0%.

With 1500 randomised to one of the 4 arms (375 per arm), assuming that 10% in the SOC only arm access clinical services, we will have 90% power to detect an increase in uptake to 22% with the addition of one intervention (peer navigator support only, or sexual health only). We will also have > 90% power to detect an increase in uptake from 22% in the arms with only one intervention, to 38% in the arm with both interventions (peer support and sexual health).

With 750 allocated to each intervention (SRH or peer navigation) and 80% follow-up, assuming no interaction between the interventions, we will have 80% power to detect a reduction in the proportion of individuals with detectable viral load from 7.0% to 3.4%, or from 5.0% to 2.0%. These calculations are technically valid for one intervention assuming the other has no effect, but may apply approximately if both are effective.

### Randomisation and assignment of intervention

Participants are randomised to one of 4 arms in a 1:1:1:1 allocation, stratified by sex and geographic region of the surveillance area. JD (Senior Data Manager) generates an eligibility list from all men and women aged 16–29 inclusive living in the areas accessible the peer navigators using the 2019 health and demographic surveillance census list. Eligibility criteria is being a male or female, aged 16–29 inclusive and living in the areas that are accessible to the area-based peer navigators (that had already been mapped). The trial statistician (NM1) generates a random sample of *n* = 3000 stratified by sex and geographical area (defined by accessibility to the area-based peer navigators). NM1 (statistician) then generates a random allocation list to one of four arms: SOC; peer navigation; STI testing; and peer navigation and STI testing. The allocation is then uploaded into the electronic data collection tool (REDCap) and is only visible after the participant consents to enrolment. Given the possibility of imbalance in informed consent by chance, monthly randomisation reports are generated to monitor balance in the number enrolled in each arm; the randomisation may be adapted if needed at the interim recruitment point.

Randomisation and Allocation are kept separate throughout. Investigators and statistician remain blinded to allocation throughout. The participants and intervention delivery teams are not blinded.

### Eligibility screening and recruitment

The study team visit the sampled individuals in their homes to invite them to participate in the study. They complete a brief eligibility screen and provide potential participants with information about the trial. HIV counselling and POCT will be offered and encouraged at baseline, through linkage to the clinical services offered to all who enrol. Accepting HIV POCT at baseline is not a condition for participation; however, to be eligible the individual must agree to being contacted at 12 months for anonymous HIV testing. Following informed consent, participants receive a unique study identifying number (ID) and study ID card. They are asked to complete a brief electronic enrolment questionnaire on a tablet. After the questionnaire is completed, the individual’s trial allocation is revealed, with a related participant information sheet for that arm.

#### Referral to the clinic for symptoms and clinical events

All participants are encouraged to visit the clinic for any medical concerns they may have during the trial. During medication refill and monitoring visits, participants on PrEP complete a standardised symptom screening questionnaire for adverse effects, as per South African clinical guidelines. All participants on PrEP receive regular creatinine tests to monitor their renal function. Participants who have severe (grade 3/4) adverse effects are referred to the clinic for medical evaluation and will be follow-up until the event is resolved.

### Data collection, management and analysis

#### Follow-up for outcome ascertainment

Clinic attendance during the trial is captured at the mobile study clinics and all the Primary Health Clinics serving the AHRI surveillance area using the participants’ unique identifying number and/or scanning the barcode with the unique identifier on the clinic referral slip. Participants who have been given a referral slip but did not bring it to the clinics are identified using an algorithm based on their unique demographic surveillance identifier number, name, date of birth, residential address, telephone number, and identity of the research assistant who recruited them in their enrolment.

All participants, irrespective of whether they initiated PrEP or ART, will be visited by the study team in their home 12 months after enrolment. Participants will fill out a questionnaire regarding their uptake and experience of HIV prevention and care services, uptake of contraception and incidence of pregnancy, mental health (using PHQ9), and quality of life. They will be asked to provide a DBS for anonymous HIV ELISA and HIV viral load testing. All participants will also be offered STI testing and offered HIV counselling and POCT, and referral to a clinical service of their choice.

#### Data management

Data will be captured electronically on tablets using REDCap software [37]. Automatic checks for invalid values, internal consistency and implausible responses will be programmed into REDCap, and additional data validation checks will be run after data collection. All changes will have an audit trail. The data from REDCap will be uploaded to a MySQL database server within a secure server cluster at AHRI.

#### Statistical analysis

All analyses will be conducted on an intention-to-treat (ITT) basis. A significance level of 0.05 will be used unless otherwise stated. For all outcomes, analyses will be adjusted for age and sex, since these are known a priori to be strongly association of HIV infection. Additional analyses adjusted for covariates that show baseline imbalance may be conducted to explore the robustness of our results.

To examine the effect of the intervention on sexually transmissible HIV at M12, we will use logistic regression to estimate the odds ratio (OR) and 95% confidence interval (CI) for effect of both delivery model (SOC vs peer navigator) and SRH package vs SOC, assuming no interaction. As a secondary analysis, we will fit a model with delivery model (SOC vs peer navigator), SRH intervention (yes/no) and their interaction which we will test. We will also report the effect of the three intervention combinations (peer navigator only, SRH only, both) vs. control (SOC alone) based on a logistic regression model. Participants who cannot be contacted at M12 will be excluded from the analysis, and missing data will not be imputed. Since the primary analyses will be adjusted for baseline variables that are strongly associated with the outcome, and no other M12 data can be collected for these participants that could be used as auxiliary variables in the imputation model, imputation is unlikely to provide any additional information.

To examine the effect of each intervention on the demand for risk-informed HIV prevention services, we will fit a logistic regression model with treatment group as a 4-level categorical variable, to estimate the OR and 95% CI for the following pairwise comparisons: SOC vs peer navigator alone; SOC vs SRH alone; SOC vs peer navigator combined with SRH. Participants who are lost to follow-up will be included in the analysis and assumed not to have linked to services.

To assess acceptability and feasibility of the intervention (the third primary objective), we will calculate the proportion and 95% confidence interval of participants who consent to participate in the trial, and of participants whose HIV viral load is collected 12 months after enrolment.

#### Adverse event reporting and management

Adverse events (AE) and serious adverse events (SAE) will be captured through clinic staff and peer navigators, as well as the process evaluation, community engagement units and community advisory boards and a hotline and will be recorded up to 18 months after the start of the intervention. Reported AEs and SAEs will be monitored, categorized based on an established grading system, and followed-up accordingly by AHRI. The study clinical monitor, based at AHRI, will review all severe AEs and all SAE to ensure follow-up and reporting. All SAEs will also be reported to Trial Advisory Group. Annual reports with full listings of SAEs will be submitted to Ethics Review Boards.

### Process evaluation

**Fig. 3:**
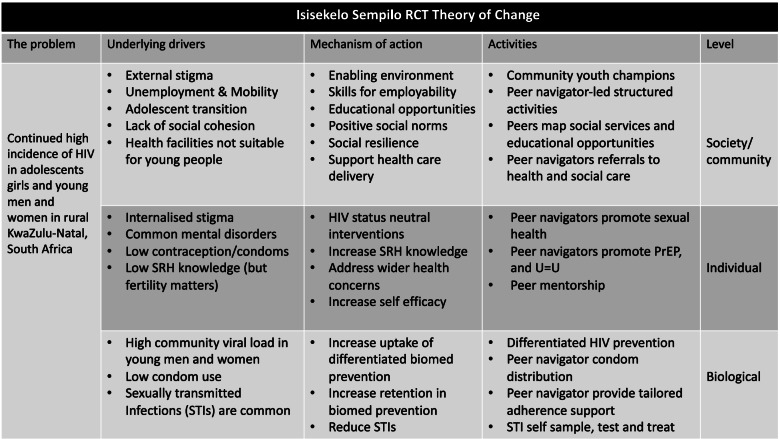
Isisekelo Sempilo RCT Theory of Change

We will conduct a process evaluation using mixed methods, including self-completed questionnaires, clinic data to quantify the uptake of each component of the intervention, and activities recorded by the intervention teams. We will conduct in-depth interviews with participants, intervention delivery teams, nurses/clinical research assistants, research assistants and peer navigators, and will conduct natural group discussions with community groups and intervention delivery teams. A satisfaction survey will be administered as part of the end-line survey. Using the intervention theory of change (Fig. 3) the process evaluation will explore topics including the acceptability/experience, feasibility, reach/coverage (for whom it worked and didn’t work) and fidelity.

### Ethics

Ethical approval has been obtained the University of KwaZulu-Natal Biomedical Research Ethics Committee (BREC/00000473/2019) and UCL Research Ethics Committee (5672/003). All staff (including peer navigators) will be provided with training on research ethics including confidentiality, voluntary participation and good clinical practice. Written informed consent will be obtained from all participants aged 18–29 years; written assent from participants aged 16–17 years, with written consent from their parents or guardian. We will establish a Trial Advisory Group with clinical trials, PrEP, statistical and social science expertise to oversee the trial. This is an effectiveness trial of different models of service delivery and all tests and drugs used are approved for clinical use in South Africa. All clinical care follows South African clinical guideline. The risk of harm is anticipated to be low.

## Discussion

Innovation in HIV prevention and treatment has outpaced the ability to implement them. The output of this trial will be a model of delivering risk-informed HIV prevention and care through comprehensive SRH for young people living in rural and semi-urban areas of South Africa. The interventions in this study have been developed through participatory research and build on the provincial plans for escalating community caregivers’ support for primary care in the context of COVID19. Moreover, we will have adapted novel methodological approaches to rapidly evaluate the efficacy, sustainability and equity of interventions to reduce prevalence of transmissible HIV in rural KwaZulu-Natal.

We will use AHRI’s Wellcome public engagement unit to ensure that this work translates into direct patient benefit and the science of evaluating complex interventions. We disseminate findings widely, using a range of media, e.g. workshops with youth and district service providers, radio, websites, international and South African AIDS conference and peer reviewed journals, reports to funders and policy makers, and through HIV prevention networks. The trial’s potential beneficiaries inlcude young people who will benefit from a more person-centred approach to HIV prevention and care; public and communities benefit from reduced HIV morbidity and mortality; and national policy makers who have called for innovations in scalable models to deliver effective HIV prevention interventions and have been engaged in this trial from its inception.

We anticipate that our trial will not only inform a large-scale evaluation of the optimised intervention but will provide South Africa and other southern Africa health policymakers with evidence for delivering ART-based HIV prevention, which aligns with the planned moves to shift care for long-term conditions into the community. This will fill an evidence gap for delivery models to tackle unmet SRH needs, including unplanned pregnancies, STIs and HIV in young people.

Trial registration Number at clinicaltrials.gov NCT04532307.

## Data Availability

All datasets generated from this study will be in the study will be presented in the final manuscript and will thereafter be made publically available through the AHRI data repository site. The full study protocol, study data collection tools and consent forms are available from the author.

## Abbreviations

AHRI: Africa Health Research Institute
SRH: Sexual and Reproductive Health
HIV: Human Immunodeficiency Virus
SAE: Serious Adverse Events
DSMB: Data Safety And Monitoring Board
IDI: In-Depth Interview
APEASE: Acceptability, Practicability, Effectiveness, Affordability
Safety/Side: Effects, Equity
SA: South Africa
KZN: KwaZulu-Natal
UKZN: University of KwaZulu-Natal
UCL: University College London
PHC: Primary Care Clinics
STI: Sexually Transmitted Infection
PrEP: Pre-Exposure Prophylaxis
UTT: Universal Test and Treat
SOC: Standard of Care
POCT: Point of Care Tests
